# Current understanding and theories of wellbeing washing in the context of workplace health and wellbeing: A scoping review protocol

**DOI:** 10.1016/j.mex.2025.103452

**Published:** 2025-06-19

**Authors:** Éadaoin Ryan, Niamh Imbusch, Mary Kinahan, Róisín Guilfoyle

**Affiliations:** a School of Management, People and Organisations, Faculty of Business, Technological University Dublin, Aungier Street, Dublin 2 D02 HW71, Ireland; b School of Business, Maynooth University, Maynooth, Co. Kildare, Ireland; c Library Services, Technological University Dublin, Park House, Grangegorman, Dublin 7 D07 EWV4, Ireland

**Keywords:** Wellbeing washing, Wellwashing, Workplace wellbeing, Scoping review, Scoping Framework for Emerging Concepts in Practitioner Literature.

## Abstract

Workplace health and wellbeing practitioners have recently voiced concerns regarding the emerging concept of wellbeing washing (WW). While practitioner literature extensively discusses this concept, research supporting the examination of it and offering solutions is lacking. This scoping review protocol outlines a means of comprehensively investigating and mapping the mainly practitioner-authored literature on WW, to support the establishment of a working definition and deeper understanding of this concept, which will inform future research.

A search strategy will be employed to map information from diverse practitioner literature sources. Guided by the JBI Scoping Review Framework, two reviewers will use predefined criteria to independently screen included sources and capture information using a standardized data extraction tool. A basic qualitative content analysis will be conducted and results presented in tabular form. A narrative summary will complement the tabulated results, providing further context.

• The scoping review will identify, map, and synthesize existing literature on the emerging concept of WW.
• The JBI Scoping Review Framework and PRISMA-ScR reporting tool will be employed, to ensure a methodical and systematic approach.
• The outcomes aim to support the establishment of a working definition and provide deeper understanding of WW to inform future research.

## Graphical abstract

**Figure:**
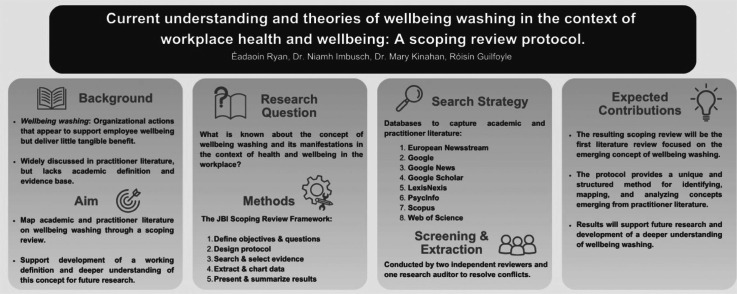

Specifications table

**Table:** 

Subject area:	Psychology
More specific subject area:	*Workplace health and wellbeing*
Name of your method:	Scoping Framework for Emerging Concepts in Practitioner Literature
Name of your protocol:	*Current understanding and theories of wellbeing washing in the context of workplace health and wellbeing: A scoping review protocol*
Reagents/tools:	*Not applicable*
Experimental design:	*Not applicable*
Trial registration:	*This scoping review protocol is registered in the Open Science Framework:*https://doi.org/10.17605/OSF.IO/NRQXT
Ethics:	*Work on this scoping review protocol did not involve human subjects, animal experiments, or data collection from social media platforms. Therefore, ethical approval and informed consent were not required.*
Value of the Protocol:	*This protocol offers a structured framework for synthesizing practitioner literature, providing a valuable methodology for identifying, mapping, and analyzing information about under-researched concepts or those emerging from practitioner literature, using wellbeing washing as a case study.* *Through establishing a systematic and rigorous approach, this protocol provides a critical starting point for understanding the concept of wellbeing washing and lays the foundations for a new area of research. The resulting scoping review will be the first literature review focused on this emerging concept.* *The protocol also provides a methodology for developing a working definition and deeper understanding of an emerging concept, using wellbeing washing as a case study.*

## Background

The concept of wellbeing washing (WW) has recently emerged as a concern in the field of workplace health and wellbeing, drawing parallels to greenwashing in discourse on the environment [1]. While no universal definition exists, the working definition in use for this protocol describes WW in the workplace as organizational actions that appear to support employee wellbeing but provide little tangible benefit, leaving employees with no meaningful improvement. Despite growing recognition amongst practitioners, the concept of WW in the workplace remains under-researched. As this is an emerging topic with little peer-reviewed academic literature explicitly addressing WW in the workplace, practitioner literature currently provides the primary source of conceptual discussion and practical insight. Accordingly, some practitioner sources that have not undergone academic peer review are referenced in this article to reflect the current state of knowledge [[2], [3], [4], [5], [6]].

Workplace wellbeing practitioners have begun addressing WW, emphasizing the risks of inadequate or superficial wellbeing initiatives, such as increased stress and burnout in employees [2,3], and reputational damage and increased employee turnover in organizations [4,5]. However, outside of one practitioner study, which found that 38 % of employees believe their employer engages in WW [6], there is little data available to substantiate these concerns. Academic research has explored related fields, such as inadequate workplace wellbeing initiatives [7,8], and the individualization of workplace wellbeing [9,10], however, academics and practitioners alike have neglected to explicitly address WW in the workplace as a distinct phenomenon. Although health and wellbeing initiatives are increasingly championed as an essential element of modern organizations, this lack of research on WW leaves its potential impacts on worker health and wellbeing, organizations, and wellbeing initiatives largely unclear.

Integrating practitioner concerns into academic discussion has the potential to enhance understanding and guide meaningful intervention development, as shown in the examples of worker burnout [11,12] and mindfulness [13], two concepts with roots in practitioner observation. In the case of WW, there is a need to build on practitioner insight through rigorous research that identifies evidence of this phenomenon. Greater examination of the concept is required to understand its characteristics, its potential impact on individuals and workplaces, and to explore possible solutions.

To explore this emerging concept in more depth, it is first necessary to examine the current literature and build an understanding of what is already known about it. A scoping review is an important tool in this endeavor, as this style of review is designed to systematically identify and map details such as key concepts, definitions, and characteristics of a particular topic. This review aims to fill a gap in knowledge by identifying, mapping, and synthesizing information on WW in the workplace. The findings may contribute to defining and understanding this concept by establishing a foundation for future research. Additionally, this review will contribute to the body of literature that acknowledges the value of incorporating practitioner knowledge and experience into academic research [14,15].

This protocol was developed to ensure a systematic and rigorous approach to the scoping review. It outlines the research questions and objectives, eligibility criteria, and methodologies including details of a search strategy, evidence source selection, data extraction, critical appraisal, and data analysis and presentation. This protocol presents a framework that may be used to investigate emerging concepts, practitioner literature, or heterogenous literature. For the remainder of this protocol, the term ‘wellbeing washing’ will apply to a workplace context only.

## Description of protocol

### Design

This scoping review protocol was designed using the guidelines outlined in the established JBI Scoping Review Framework [16], as well as guidance provided in the JBI Protocol Template which accompanies their Framework. It also uses the Population, Concept, Context (PCC) framework described by JBI [16].

This protocol focuses on the first three stages of the JBI Scoping Review Framework including (1) defining and aligning the objectives and questions, (2) developing and aligning the inclusion criteria with the objectives and questions, and (3) describing the planned approach to evidence searching, selection, data extraction, and presentation of the evidence. The scoping review will then follow the remaining stages of this framework, to include (4) searching for the evidence, (5) selecting the evidence, (6) extracting the evidence, (7) analysis of the evidence, (8) presentation of the results, and (9) summarizing the evidence in relation to the purpose of the review, making conclusions and noting any implications of the findings [16, p. 412]. To enhance this systematic approach, the Preferred Reporting Items for Systematic Reviews and Meta-Analyses extension for Scoping Reviews (PRISMA-ScR) checklist will be employed to report the review results [17]. This structured approach using established frameworks can reduce bias, while enhancing reproducibility and rigor [18,19]. Prior to the development of this protocol, a search was conducted for existing scoping and systematic reviews on the topic of wellbeing washing. The Cochrane Database of Systematic Reviews, JBI Evidence Synthesis, and Google Scholar databases did not identify any existing or in-progress systematic or scoping reviews on this concept.

### Review questions and objectives

The primary research question of the review is: *What is known about the concept of wellbeing washing and its manifestations in the context of health and wellbeing in the workplace?* This question aligns with the exploratory nature of scoping reviews, allowing for a comprehensive exploration of a variety of sources. This style of question, with a large scope asking what is known about a specific topic, has been used in other scoping reviews to guide broad explorations [[20], [21], [22]].

To build a clearer understanding of this concept, the following sub-questions were identified and will guide data extraction and analysis:

• RQ1: How is wellbeing washing defined in the literature?
• RQ2: What key characteristics are used to identify wellbeing washing?
• RQ3: What examples of wellbeing washing have been discussed?
• RQ4: What are the reported impacts of wellbeing washing on workers, management, and organizations?
• RQ5: What are the reasons that wellbeing washing occurs?
• RQ6: What solutions have been proposed to address wellbeing washing?
• RQ7: What evidence exists to support claims of wellbeing washing?

The primary objective of this scoping review is to comprehensively explore the literature on wellbeing washing (WW). This will involve identifying, charting, and analyzing evidence of WW, including possible definitions, characteristics, identifying criteria, patterns, examples, and potential impacts of this phenomenon, as well as any data gathered through primary research. The term ‘evidence’ is used here to refer to information derived from sources that contribute to an understanding of WW. The aim of this review is to map out what is currently known about WW and, through collating, summarizing and analyzing the evidence, to contribute to a deeper understanding of the concept. Additionally, this work seeks to support future research and discourse on WW as an emerging concept.

### Eligibility criteria

The eligibility criteria were designed to be as broad as possible, to align with the objective of a wide-ranging investigation of the concept of WW and to facilitate the inclusion of evidence from heterogenous practitioner literature sources, which may appear in formats or publications that are not typically incorporated into academic literature reviews.

#### Population

Sources must discuss WW in relation to workers, management, or organizations within a workplace setting. There will be no restrictions on age, gender, location, or other demographic characteristics of participants. Sources focused on non-workplace contexts or populations outside of a workplace environment will be excluded.

#### Concept

Sources must address WW within the context of the workplace. For the purposes of this review, WW can be considered to occur when organizations appear to invest in improving employee health and wellbeing, but these workers perceive little to no tangible benefit or meaningful improvement. Sources that do not directly address the concept of WW, or those that discuss wellbeing in the workplace without reference to this concept, will be excluded.

#### Context

Sources must specifically discuss WW in workplace settings. There will be no restrictions based on the geographic location, industry, sector, or type of workplace. Sources discussing WW outside of a workplace context or unrelated to employee perceptions of organizational health and wellbeing initiatives will be excluded.

#### Types of sources

The review will accept a broad range of source types including, but not limited to:

• Grey literature: Government reports, industry research, websites of workplace well-being practitioners, blog posts.
• Commercial literature: Newspaper articles, trade journals, industry magazines.
• Academic literature: Journal articles, book chapters, conference proceedings, and PhD theses.

Further source types may be included, depending on what is uncovered during the search. There will be no limitations on publication date.

Sources must be written in English and relevant to the concept of WW as defined in the protocol. Sources that are unrelated to the discourse on WW, or do not provide insights into the concept of WW, will be excluded.

### Search strategy

The search strategy was developed to identify sources with direct references to the terms ‘wellbeing washing’, ‘wellwashing’, and ‘wellness washing’. This approach ensures the collection of evidence directly related to the concept of WW. Extensive preliminary test searches conducted ahead of protocol development did not identify any additional terms or related fields that were useful in capturing sources on WW. As the objective of the review is to identify sources specifically on WW, expanding the search to broader terms and phrases was deemed inappropriate.

As WW has primarily been discussed in practitioner literature and media sources, it is essential that the databases used in the search will capture information from both practitioner as well as academic sources. To achieve this, the selected databases allow for access to a diverse range of sources from practitioner, grey, and commercial literature, as well as academic articles from the fields of workplace wellbeing, workplace health promotion, organizational psychology, and human resource management.

This search strategy was developed in consultation with a research support librarian to ensure an informed approach to database selection and search term development. Table 1 outlines the selected databases and corresponding query strings that will be used in the search.

**Table 1:** Selected databases and query strings.

Database	Search Query
1. European Newsstream	("wellwashing" OR "wellness washing" OR "well-being washing" OR "wellbeing washing")
2. Google	("wellwashing" OR "wellness washing" OR "well-being washing" OR "wellbeing washing")
3. Google News	("wellwashing" OR "wellness washing" OR "well-being washing" OR "wellbeing washing")
4. Google Scholar	("wellwashing" OR "wellness washing" OR "well-being washing" OR "wellbeing washing")
5. LexisNexis	("wellwashing" OR "wellness washing" OR "well-being washing" OR "wellbeing washing")
6. PsycInfo	("wellwashing" OR "wellness washing" OR "well-being washing" OR "wellbeing washing")
7. Scopus	("wellwashing" OR "wellness washing" OR "well-being washing" OR "wellbeing washing")
8. Web of Science	("wellwashing" OR "wellness washing" OR "well-being washing" OR "wellbeing washing")

Results from the Google database search will be limited to the first 100 results. This decision was made to ensure the inclusion of only the most relevant results. A backward citation search will also be conducted to identify additional sources cited within the included studies.

### Search validation procedure

Preliminary test searches were conducted to assess the effectiveness of the search strategy in capturing relevant sources, to identify and refine search terms, and to adjust Boolean operators. When the search strategy is executed, an independent verifier will replicate the search strategies across each database, to compare and confirm results. Any discrepancies between these results will be reviewed, and adjustments will be made where necessary to ensure consistency.

### Source of evidence selection

Following search strategy execution, results will be collated and screened for duplicates using Mendeley Reference Management software. Once duplicates are removed, the titles and abstracts of remaining sources will be manually screened against the inclusion and exclusion criteria in a two-stage process. In alignment with guidelines from JBI, two screeners will work independently to screen all included sources [16,23]. To ensure consistency and alignment with the eligibility criteria, software use, and steps in the process, pilot testing will be conducted by both screeners before each stage.

The first screen will review titles, abstracts, and metadata. Where an abstract is unavailable, a brief review of source content will be conducted instead. The second screen will involve a full-text review of all included sources. After each stage, the results from both screeners will be compared and discrepancies discussed. An independent research auditor will be consulted in cases where consensus cannot be reached on the inclusion or exclusion of a source. Reasons for exclusion of a source after a full-text screen will be documented and reported in the scoping review, along with details of the complete results of the search and the source inclusion process. A PRISMA-ScR flow diagram will be used to illustrate the screening and inclusion/exclusion process [17].

### Data extraction

Following the principles of data extraction outlined in JBI scoping review guidance [24], two extractors will independently extract data from all included sources. Data will be extracted manually from each included source using a custom-designed data extraction tool in Microsoft Excel, developed specifically for this review. This standardized tool was designed to capture descriptive information relevant to the research questions outlined in this protocol, which may help to support the development of a definition and deeper understanding of WW. Preliminary searches suggested that this review is unlikely to capture academic studies of WW, given the dearth of academic literature on this topic. Consequently, the data extraction tool has been tailored to capture information from commercial and grey literature sources that discuss practitioner experiences and insights on WW. Table 2 outlines the data that will be extracted using this tool.

**Table 2:** Data fields for extraction.

Category	Data Fields
Descriptive Information	Title, Author(s), Source Title, Publication Date, Associated Organization, Source Type, URL, Country of Publication, Organization Under Discussion, Demographic Under Discussion, Description of Evidence Presented, Tone or Bias, Main Objective of Source, Limitations

Wellbeing Washing Details	Definitions of Wellbeing Washing, Key Words/Terms, Characteristics, Specific Examples/Case Studies, Stakeholders, Practices/Strategies Described as Wellbeing Washing, Reasons for Wellbeing Washing, Reported Impacts on Employees, Reported Impacts on Management, Reported Impacts on Organizations, Other Outcomes and Effects, Implications, Proposed Solutions, Notable Quotations

As data extraction is a flexible and iterative process [24], modification or streamlining of the extraction tool may be necessary during the extraction process, particularly during pilot testing. Any modifications will be reported in the final review, to ensure transparency. Before extraction commences, both extractors will pilot test and discuss the data extraction tool. This will ensure that relevant data will be captured and that there is consensus and clarity on the use of the tool. Once the form and process have been finalized, extractors will proceed independently, working in parallel to extract data from all included sources. When extraction is complete, data from both extractors will be merged and reviewed to ensure consistency and agreement on what has been extracted. An independent adjudicator will be consulted to make a final decision in cases where consensus cannot be reached.

### Data analysis and presentation

As scoping reviews aim to collect and chart a wide variety of information on a particular topic, in-depth analysis and synthesis of results are not generally conducted [16]. Considering this, a basic qualitative content analysis will be conducted, in alignment with recommendations from JBI on scoping reviews designed to identify the key characteristics and factors of a particular concept [24]. An inductive approach will be adopted, allowing for the development of an understanding of WW through the analysis of extracted information such as definitions and characteristics of this concept. Extracted data will be coded and analyzed using NVivo software.

Extracted data will be presented in the following manner:

• Tables will be used to organize, present, and summarize key findings such as definitions, characteristics, reported impacts, and proposed solutions.
• A narrative summary will contextualize the findings in relation to the research questions and objectives, highlighting key themes, insights, and relevant patterns.
• Quantitative descriptive statistics will be used to summarize information such as source type, frequency of characteristics, prevalence of definitional themes, and other notable insights identified during analysis.

### Critical appraisal

Critical appraisal of sources is not generally required in a scoping literature review, as the primary aim of this review type is to map and describe evidence rather than assess its quality [16,25]. However, where there is a clear rationale for an appraisal, it is possible to conduct one [16,17], and it is proposed to conduct one in this instance.

Prior to the development of this protocol, preliminary database searches on WW indicated that a significant amount of the available information on this concept originates from practitioner literature such as news articles, reports, and websites. Consequently, it is anticipated that this review will capture sources mostly from such literature, rather than from academic research. To assess the rigor and quality of this evidence, a critical appraisal will be conducted on all the included sources. In line with the JBI Scoping Review Framework, this appraisal process will not serve as a basis to include or exclude sources of evidence, but only as a method of evaluation of included sources. The aim of this appraisal process is to examine the type and quality of the evidence for WW, as well as its limitations, strengths, and biases. While unusual for a scoping review, this process may help identify gaps that highlight the need for more rigorous academic research in this area.

The Authority, Accuracy, Coverage, Objectivity, Date, Significance evaluation framework (AACODS), developed by Tyndall [26], will be used to guide the critical appraisal process. This framework was designed to evaluate the reliability and quality of sources in grey literature and is particularly relevant for assessing sources based on expert opinion. It is loosely based on the JBI model of evidence, which acknowledges that knowledge formed from “opinion, experience and expertise” [27, p. 211] is a valid form of evidence, especially where evidence from academic research is unavailable. It was designed out of the need for a tool to critically evaluate grey literature, with enough flexibility to be used on heterogenous source types.

As it is anticipated that the majority of included sources in this review will be from grey and commercial literature, and difficult to assess using more traditional tools designed for appraising academic studies, the AACODS provides a structured yet adaptable framework for assessing their quality. While originally designed for grey literature, it is proposed that this flexible framework is well-suited to commercial literature which can share similarities with grey literature, including a lack of peer review, heterogenous source types on similar topics, and the use of expert knowledge as a source of evidence. This makes it a suitable and flexible tool for conducting critical appraisal in the context of this scoping review, which is expected to capture a diverse range of source types from both commercial and grey literature.

### Protocol validation

To ensure the validity of this *a priori* protocol, it was developed using established guidance from JBI, including three steps of the JBI Scoping Review Framework: (1) defining and aligning the objectives and questions, (2) developing and aligning the inclusion criteria with the objectives and questions, and (3) describing the planned approach to evidence searching, selection, data extraction, and presentation of the evidence [16, p. 412]. The use of this framework and the PRISMA-ScR guidelines supports a systematic and reproducible approach. Additionally, preliminary test searches were conducted to ensure that the search strategy and selected databases were appropriate and would capture sources aligned with the review objectives. These searches resulted in the selection and refinement of search terms and Boolean operators, to improve their precision and ensure that the strategy would capture relevant literature. To further enhance the validity of the review, the data extraction process will be pilot tested on included sources and modified where necessary to ensure the capture of data relevant to the research questions and objectives. To promote transparency, an early version of this protocol was published on the Open Science Framework [28]. Although not peer-reviewed, this registration enhances methodological transparency.

## Limitations

While great care has been taken in the development of this protocol to minimize limitations, there are some that should be acknowledged. The Google database search will be restricted to the first 100 results. While this choice was made to ensure that only the most relevant results are included, it may mean that some sources are excluded from the review. Additionally, only English-language sources will be included, which means that sources in other languages may be overlooked. These limitations highlight potential areas for future research, which could further expand knowledge on the concept of WW.

## CRediT authorship contribution statement

**Éadaoin Ryan:** Conceptualization, Methodology, Validation, Writing – original draft, Writing – review & editing, Visualization, Funding acquisition. **Niamh Imbusch:** Conceptualization, Methodology, Writing – review & editing, Supervision, Project administration, Funding acquisition. **Mary Kinahan:** Conceptualization, Methodology, Writing – review & editing, Supervision, Funding acquisition. **Róisín Guilfoyle:** Conceptualization, Methodology.

## Declaration of competing interest

The authors declare that they have no known competing financial interests or personal relationships that could have appeared to influence the work reported in this paper.

## Data Availability

No data was used for the research described in the article.
